# The Bustelo Bolus: Caffeine Withdrawal Mimicking Status Migrainosus in a Patient With Systemic Lupus Erythematosus and Systemic Sclerosis Overlap

**DOI:** 10.7759/cureus.109884

**Published:** 2026-05-29

**Authors:** Thomas C Landry, Youjin Kim, Sylvia Riley

**Affiliations:** 1 Internal Medicine, Legacy Salmon Creek Medical Center, Vancouver, USA; 2 Internal Medicine, Vancouver Clinic, Vancouver, USA

**Keywords:** caffeine withdrawal headache, coffee consumption, inpatient diagnostic stewardship, status migrainosus, stevens-johnson syndrome (sjs)

## Abstract

Caffeine is the most widely consumed psychoactive substance in the world, and abrupt cessation in habitual users produces a stereotyped withdrawal syndrome whose dominant feature is headache. Although caffeine withdrawal is a familiar diagnosis in ambulatory and perioperative practice, it is easily overlooked in hospitalized patients on a complex medical service, where the differential for new headache is dominated by autoimmune, infectious, and vascular etiologies. We report a 33-year-old woman with overlap connective tissue disease (systemic lupus erythematosus, mixed connective tissue disease, and systemic sclerosis) admitted for doxycycline-associated Stevens-Johnson syndrome and oral mucositis. On hospital day 3, she developed an excruciating bilateral throbbing headache with photophobia, phonophobia, and nausea. Magnetic resonance imaging of the brain and magnetic resonance angiography of the head were unremarkable apart from non-specific subcortical white matter foci. The headache was refractory to six acute migraine therapies and to a status migrainosus protocol, and a lumbar puncture was being prepared. After ingestion of approximately 8 oz of brewed black coffee from the nursing station, the headache resolved within 30 minutes and did not recur, and the planned lumbar puncture was deferred. The patient was discharged the same day. The presentation met the International Classification of Headache Disorders, 3rd edition, criteria for caffeine withdrawal headache. The case illustrates how an inexpensive caffeine challenge can serve as both a diagnostic and a therapeutic step before invasive workup in a hospitalized patient whose habitual oral caffeine intake has stopped abruptly. Routine intake history, including caffeine, should be elicited at admission for any patient anticipated to be nil per os or to have impaired oral intake.

## Introduction

Caffeine is consumed by approximately 90% of US adults, with a mean per-capita intake of approximately 169 mg per day [1]. In habitual consumers, abrupt cessation reliably produces a withdrawal syndrome with headache as the dominant symptom in roughly half of cases, with typical onset 12-24 hours after the last dose and peak intensity at 20-51 hours [2]. The International Classification of Headache Disorders, 3rd edition (ICHD-3), defines caffeine withdrawal headache (8.3.1) as a headache developing within 24 hours after interruption of regular consumption of more than 200 mg of caffeine per day for more than two weeks that resolves spontaneously within seven days of abstinence or within one hour of caffeine ingestion of at least 100 mg [3]. Although mean intake lies below the ICHD-3 threshold of more than 200 mg per day, a substantial proportion of habitual consumers exceed this level and are at risk for withdrawal upon abrupt cessation. Substance withdrawal headaches, of which caffeine, estrogen, and opioid withdrawal are the most common, are well described in the neurology literature [4].

The diagnosis is easily overlooked in inpatient medicine. A new severe headache in a patient with established autoimmune disease typically prompts a workup directed at lupus headache, neuropsychiatric systemic lupus erythematosus (SLE), aseptic meningitis, reversible cerebral vasoconstriction syndrome (RCVS), posterior reversible encephalopathy syndrome (PRES), subarachnoid hemorrhage, and idiopathic intracranial hypertension or hypotension [5,6]. Hospitalization itself can precipitate caffeine withdrawal through nil per os orders, perioperative fasting, mucositis, dysphagia, or a simple lack of access to the patient’s habitual beverage. Perioperative survey data demonstrate that 22% of habitual caffeine consumers develop postoperative headaches compared with 7% of non-consumers and that perioperative caffeine consumption reduces this incidence [7]. Caffeine supplementation has been proposed as a routine component of enhanced recovery after surgery pathways for the same reason [8]. The same risk applies to medical inpatients whose oral intake stops abruptly, but a caffeine history is rarely a structured part of the medical admission.

We present a case in which severe refractory inpatient headache, ultimately consistent with ICHD-3 caffeine withdrawal headache, mimicked status migrainosus (a debilitating migraine attack lasting more than 72 hours despite treatment) and prompted preparation for lumbar puncture in an immunosuppressed patient with cutaneous and mucosal disease. We describe the clinical reasoning, the standard inpatient migraine and status migrainosus pathway that the team had exhausted, and the diagnostic and therapeutic value of an inexpensive caffeine challenge. This article was previously presented as a meeting abstract at the American College of Physicians Internal Medicine Meeting 2026 on April 17, 2026.

## Case presentation

A 33-year-old woman with overlap connective tissue disease, comprising SLE, mixed connective tissue disease (MCTD), and systemic sclerosis with inflammatory arthritis and Raynaud phenomenon, presented to the emergency department with a recurrent blistering rash and oral mucosal lesions five days after starting a course of oral doxycycline for a presumed minor skin infection. The serologic profile included an antinuclear antibody titer of 1:1280, hypocomplementemia, and positive U1-RNP, double-stranded DNA, Smith, and rheumatoid factor antibodies, with negative SSA, SSB, tissue plasminogen activator, and Scl-70 antibodies. She had a documented history of Stevens-Johnson syndrome (SJS) on prior exposure to doxycycline and other agents and a Schatzki ring with prior dilations and baseline solid-food dysphagia. She had been off all immunosuppressive therapy, including azathioprine, hydroxychloroquine, and prednisone, for approximately two months prior to admission because of a lapse in rheumatology follow-up. She worked as a respiratory therapist on shift schedules and reported substantial daily coffee consumption by self-report, characterized as several cups of strong coffee daily; exact milligram intake was not quantified during admission, though. Based on the typical caffeine content of dark-roast brewed coffee of approximately 100 to 200 mg per 8 oz cup, her reported intake corresponds to an estimated 300-500 mg per day.

Examination at admission demonstrated blistering and dusky lesions on the bilateral upper extremities, greater than the lower extremities and the abdomen, with oral mucosal involvement, lip crusting, and a small lateral tongue laceration. Total body surface area involvement was less than 10%. There was no ocular involvement. Vital signs were temperature 97.9°F, blood pressure 126/79 mmHg, heart rate 83 beats per minute, respiratory rate 18 breaths per minute, and oxygen saturation 100% on room air. Initial laboratory studies were notable for white blood cell count 2.0 × 10⁹/L (reference range 4.5-11.0 × 10⁹/L), hemoglobin 9.1 g/dL (reference range 12.0-15.5 g/dL; consistent with the patient’s known chronic microcytic anemia), creatinine 0.66 mg/dL (reference range 0.6-1.1 mg/dL), albumin 4.2 g/dL (reference range 3.5-5.0 g/dL), and hepatic function panel within reference limits. Concurrent with the rash, she reported a new constant bilateral band-like frontal headache rated 5/10, partially relieved by acetaminophen, without any prior history of migraine or chronic headache. Because of mucositis and pre-existing dysphagia, oral intake was negligible from admission onward.

She was admitted to the internal medicine service with a diagnosis of doxycycline-associated SJS-toxic epidermal necrolysis spectrum and started on pulse-dose intravenous methylprednisolone at 125 mg every six hours [9]. Cutaneous and mucosal findings improved over the first 48 hours. On hospital day 3, the headache escalated abruptly from 5/10 to 10/10, throbbing and bilateral, with new photophobia, phonophobia, hyperacusis, severe nausea, and emesis. Examination demonstrated no focal deficits, no nuchal rigidity, no Brudzinski sign or Kernig sign, intact cranial nerves, normal sensorimotor function, no pulsatile tinnitus, and no visual obscurations.

The differential diagnosis was broadened to include status migrainosus, lupus headache, neuropsychiatric SLE, aseptic meningitis (drug-induced or autoimmune), RCVS, PRES, subarachnoid hemorrhage, idiopathic intracranial hypertension, spontaneous intracranial hypotension, and steroid pulse-induced headache. Magnetic resonance imaging (MRI) of the brain with and without contrast and magnetic resonance angiography (MRA) of the head demonstrated several scattered T2/fluid-attenuated inversion recovery (FLAIR) hyperintense foci in the right frontoparietal subcortical white matter at the level of the lateral ventricles (Figure 1) and in the bilateral superior frontal subcortical white matter near the vertex (Figure 2), somewhat greater than expected for age but non-specific. There was no acute infarction, hemorrhage, mass, abnormal enhancement, vessel irregularity, or dural sinus thrombosis. Such non-specific subcortical white matter foci are recognized as incidental findings in young patients with connective tissue disease and are not specific for any acute pathology.

**Figure 1:**
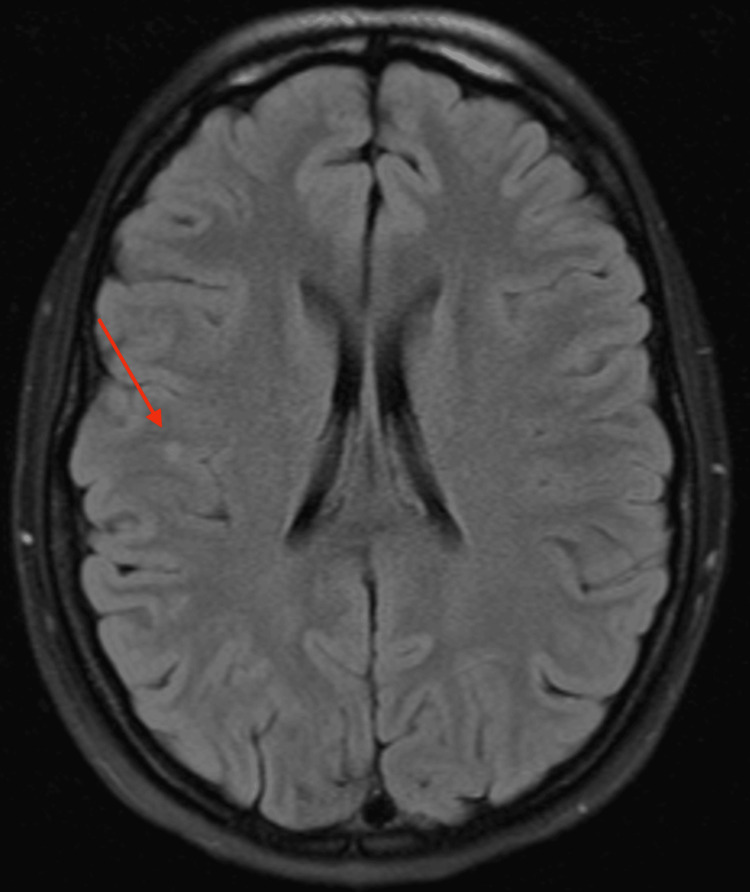
Axial T2 FLAIR sequence, brain MRI, at the level of the lateral ventricles. Hyperintense foci in the right frontoparietal subcortical white matter (red arrow). FLAIR: fluid-attenuated inversion recovery; MRI: magnetic resonance imaging.

**Figure 2:**
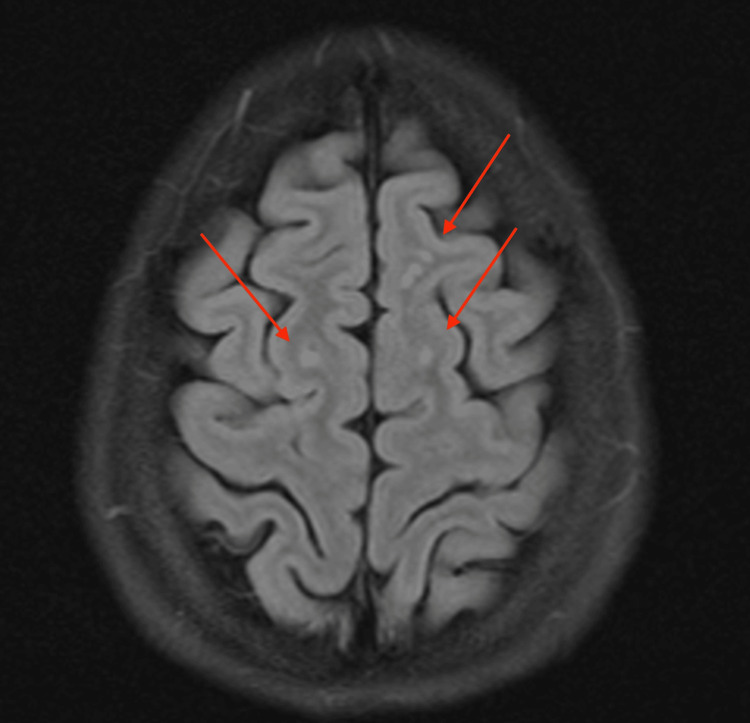
Axial T2 FLAIR sequence, brain MRI, high superior frontal level near the vertex. Scattered hyperintense foci in the bilateral superior frontal subcortical white matter (red arrows). FLAIR: fluid-attenuated inversion recovery; MRI: magnetic resonance imaging.

The team initiated a standard inpatient migraine pathway in line with current guidance for status migrainosus and acute migraine, including intravenous fluids, intravenous magnesium sulfate (2 g), ketorolac (30 mg), prochlorperazine (5 mg), sumatriptan (6 mg) subcutaneously, metoclopramide (10 mg), intravenous acetaminophen (1 g), oxycodone (5-10 mg), and lorazepam [5,10]. Relief was marginal and short-lived. Neurology consultation on hospital day 5 recommended escalation per a status migrainosus protocol, including intravenous valproate (1 g) after pregnancy exclusion, additional intravenous magnesium and prochlorperazine with diphenhydramine, transition from methylprednisolone to dexamethasone as part of the recommended escalation strategy, and lumbar puncture with measurement of opening and closing pressures, cerebrospinal fluid cell count, differential, protein, and glucose to evaluate for intracranial hypertension or hypotension and aseptic meningitis [5].

At the moment of decision, there were no red-flag features requiring immediate neuroaxial evaluation: no fever, no focal neurologic deficits, no nuchal rigidity, no postural component, and no thunderclap onset, although severity was high. Prior to initiating valproate or proceeding to lumbar puncture, the team revisited the patient’s intake history and confirmed habitual high daily caffeine consumption with abrupt cessation since the onset of mucositis four days earlier. The patient was offered approximately 8 oz of brewed coffee from the nursing station (Cafe Bustelo, a concentrated dark roast). Within approximately 30 minutes, she reported near-complete resolution of headache, nausea, photophobia, and phonophobia. The headache did not recur. She was discharged the same day on a tapered course of oral prednisone for SJS, with instructions to resume habitual caffeine intake, ophthalmology follow-up, and rheumatology re-engagement. Telephone follow-up at one week confirmed sustained resolution. The doxycycline-SJS attribution was supported by appropriate temporal exposure, mucocutaneous phenotype, dechallenge response to systemic corticosteroids, and a documented prior Stevens-Johnson event after doxycycline rechallenge, with no plausible alternative drug exposure during the current admission window.

Written informed consent was obtained from the patient for publication of this case report and any accompanying images.

## Discussion

A caffeine withdrawal headache (ICHD-3 8.3.1) is a clinical diagnosis. The patient met all four criteria: regular consumption of more than 200 mg of caffeine per day for more than two weeks by patient self-report; abrupt cessation; headache developing within 24 hours of cessation; and resolution within one hour of caffeine ingestion of at least 100 mg [2,3]. The temporal profile of the case is also consistent with the empirically validated natural history of caffeine withdrawal: onset within the first 24 hours, peak between 20 and 51 hours, and a duration of two to nine days when untreated [2]. The patient’s headache began on the day of admission, escalated through hospital day 3, and persisted until caffeine was reintroduced on hospital day 5.

The proposed mechanism is incompletely understood but classically attributed to changes in cerebral perfusion mediated by adenosine signaling on the trigeminovascular system. Caffeine is a competitive antagonist of adenosine A1 and A2A receptors, and chronic exposure leads to receptor upregulation [11]. Abrupt cessation removes the antagonism and allows endogenous adenosine to bind upregulated receptors, producing cerebral vasodilation that is detectable on transcranial Doppler as increased blood flow velocities; reintroduction of caffeine reverses these changes within 30 minutes, with a return to baseline velocities by approximately two hours [12]. More recent reviews argue that this purely vascular model is insufficient to explain prodromal and non-headache symptoms of withdrawal and implicate hypothalamic and dopaminergic mechanisms in addition [13,14]. Beyond vasodilation, adenosine-receptor upregulation may also enhance trigeminal nociception and central sensitization, which helps explain the migraine-like phenotype [13,15]. The clinical relevance is that caffeine withdrawal headache phenotypically overlaps with migraine, including throbbing quality, photophobia, phonophobia, and nausea, which is part of why it is a faithful migraine mimic in habituated patients [13,15].

The differential diagnosis was reasonably anchored on autoimmune and vascular etiologies. The patient had untreated SLE/MCTD/scleroderma overlap, recent steroid pulse, leukopenia, non-specific subcortical white matter lesions on MRI, and abrupt escalation in pain. Lupus headache, neuropsychiatric SLE, aseptic meningitis (drug-induced or autoimmune), RCVS, PRES, intracranial hypertension or hypotension, and steroid-induced headache from high-dose intravenous methylprednisolone were each reasonable considerations and are addressed in current rheumatology and inpatient headache guidance [5,6]. RCVS in the setting of caffeine withdrawal has itself been reported [16]. Our case differs in that no MRA-detectable vasoconstriction was observed and the clinical phenotype was a status migrainosus mimic rather than RCVS, although both are consistent with adenosine-mediated cerebrovascular instability. The MRI and MRA in this case excluded acute structural and macrovascular pathology but did not exclude all of these entities, which is why the team was preparing to proceed to lumbar puncture per current status migrainosus and inpatient headache pathways [5,10].

The case illustrates a reasoning pattern well described by the dual process theory of clinical cognition: in a patient with a complex problem list, attention is drawn to the rare and dangerous diagnoses on the differential, and common physiologic dependencies are deprioritized [17]. The features of this case were a habitual high-intake caffeine consumer, a respiratory therapist on shift schedules, an abrupt and sustained interruption of intake driven by mucositis and pre-existing esophageal dysphagia from a Schatzki ring, and a hospital environment without ad hoc beverage access. None of these features were elicited as part of the structured admission history. The headache was instead managed within the inpatient migraine framework, which is appropriate but not sufficient when caffeine withdrawal is the underlying physiology.

Hospitalized patients are at structurally elevated risk of caffeine withdrawal. The literature is dominated by perioperative reports. A 1993 perioperative survey showed that 22% of habitual daily caffeine consumers developed postoperative headache compared with 7% of non-consumers, and that those who took caffeine on the day of surgery had a lower incidence of postoperative headache than those who abstained [7]. A more recent narrative review in the enhanced recovery after surgery literature has called for routine perioperative caffeine replacement [8].

A small number of inpatient case reports describe analogous presentations on medical and burn services, including a published case of RCVS attributed to caffeine withdrawal during a burns admission [16]. The medicine ward, however, lacks an analog to the perioperative enhanced recovery protocol. Patients admitted with mucositis, as in SJS or chemotherapy-induced mucositis, severe odynophagia, prolonged fasting for procedural workup, or simple preference for hospital provision over outside beverages are all at risk. Few reports have described caffeine withdrawal mimicking status migrainosus in general medical inpatients, and fewer still describe a planned lumbar puncture deferred after a therapeutic caffeine trial.

The diagnostic-stewardship implication is straightforward. A small therapeutic challenge with caffeine is low-risk, low-cost, and rapidly informative when the diagnosis is in the differential. Resolution within one hour after ingestion of at least 100 mg of caffeine is itself a diagnostic criterion under ICHD-3 [3]. Compared with the sequential cost and risk profile of valproate, dexamethasone, and lumbar puncture in an immunosuppressed patient with cutaneous and mucosal compromise, a caffeine trial has a substantial, favorable asymmetry. This case suggests that a structured caffeine history and therapeutic caffeine trial may be valuable in selected hospitalized patients with new severe headaches and abrupt interruption of habitual caffeine intake, particularly when initial evaluation excludes emergent neurovascular pathology [7].

This case has limitations. First, the diagnosis is supported by clinical criteria and a positive challenge, but the temporal coincidence with steroid taper, dermatologic improvement, and resolution of mucositis cannot be excluded as contributors. Caffeine withdrawal symptoms typically resolve within seven days, even without treatment [2]. The resolution within approximately 30 minutes of caffeine ingestion, which fell well within the ICHD-3 one-hour window, is difficult to attribute to the steroid taper or to improving mucositis, since spontaneous resolution occurs over days rather than minutes; the time course favors caffeine repletion as the specific cause. Second, pre-admission caffeine intake was characterized qualitatively by patient report and was not quantified in milligrams, and the caffeine dose delivered by the therapeutic challenge was not measured, although an 8-oz cup of dark roast brewed coffee, such as Cafe Bustelo, typically contains 100-200 mg, meeting or exceeding the ICHD-3 resolution threshold of at least 100 mg. Both caffeine figures are estimates rather than direct measurements, but the qualitative profile of several cups of strong brewed coffee daily comfortably exceeds the 200 mg per day floor.

Third, the absence of papilledema documentation and the deferral of lumbar puncture, with no opening pressure obtained, means that idiopathic intracranial hypertension and aseptic meningitis cannot be definitively excluded, though the pattern of resolution argues strongly against both. Fourth, this is a single-patient observation; the strength of the recommendation rests on the consistency of the pattern with established literature on caffeine withdrawal physiology rather than on novel mechanistic evidence.

## Conclusions

A caffeine withdrawal headache is a common, benign, and reversible cause of severe headache that can mimic status migrainosus and intracranial pathology in hospitalized patients. The diagnosis hinges on a structured intake history that is rarely a default element of the medical admission. In a habitual consumer whose oral intake has stopped abruptly, a low-dose therapeutic caffeine challenge is a safe, inexpensive, and rapidly informative step before invasive workup. Routine documentation of habitual caffeine consumption at admission, particularly for patients expected to be nil per os or to have impaired oral intake, may help prevent avoidable morbidity, hospital length of stay, and procedural risk.
